# US medical student perspectives on asian american patient inclusion in medical education: a qualitative Study

**DOI:** 10.1186/s12909-022-03550-0

**Published:** 2022-06-22

**Authors:** Thomas K. Le, Hursuong Vongsachang, Sharon Pang, George Q. Zhang, Taibo Li, Jason T. C. Lee, Shari M. Lawson

**Affiliations:** Department of Gynecology and Obstetrics, Johns Hopkins School of Medicine, 733 N. Broadway, Miller Research Building Suite 137, MD 21205 Baltimore, US

**Keywords:** Asian American, Cultural humility, Cultural competency, Medical education

## Abstract

**Background:**

Asian American (AsAm) representation is lacking in conversations surrounding cultural humility in healthcare. We aimed to investigate US medical student perspectives on AsAm patient inclusion in cultural humility training in medical education.

**Methods:**

This qualitative study analyzed free-text responses to an optional, open-ended question presented at the conclusion of an online survey assessing medical student experiences with and perceptions regarding AsAm patients in their medical education. This survey was distributed to a convenience sample of nine US medical schools. Medical students who completed at least one clinical rotation were eligible to participate in the survey. Qualitative analysis of free-text responses was conducted in an iterative process to generate emergent themes.

**Results:**

There was a total of 195 optional free-text responses from 688 participants (28%). Motivation to learn about AsAm population included shared identity and desire to better serve the AsAm population in their local community and future careers. Topics of interest included healthcare-related cultural preferences, healthcare delivery strategies, and health disparities for the AsAm population and other minority patients. Students reported that they drew on personal experiences and some pre-clinical or clinical exposures to learn about AsAm patients. Respondents cited the lack of exposure in the medical school curriculum and clinical experiences as the main challenge to learning about AsAm health and provided suggestions for the delivery of this education in their pre-clinical and clinical education. Respondents emphasized that AsAms are treated as a monolith in medical education and healthcare, despite their heterogeneity.

**Conclusions:**

Medical students identified a need and interest for greater inclusion of AsAm topics in medical education on cultural humility and minority health.

## Introduction

The population of Asian Americans (AsAms) grew by 81% between 2000 and 2019 from 10.5 million to a record 18.9 million, making them the fastest growing ethnic group in the US [1]. By 2055, AsAms are projected to become the largest immigrant group in the country [2]. In addition, AsAms are a diverse and heterogeneous group, comprising over 50 ethnic groups and speaking over 100 languages [3]. This diversity in culture and language results in a unique set of health and healthcare disparities by AsAm ethnic subgroups [4–7], yet AsAms are one of the most understudied racial/ethnic minority groups in the US [8, 9]. In medical education, cultural humility is increasingly recognized as a valuable skill to address healthcare challenges faced by minority patients [10]. However, there is a distinct lack of AsAm representation in medical education conversations about health equity and cultural humility [11].

While prior studies have examined patient perceptions of AsAm providers [12], few have looked at AsAm patients and their health disparities within medical education. A previous study conducted by our group aimed to understand medical student factors associated with readiness to care for AsAm patients [13], which is of particular importance as studies demonstrate that graduating medical students feel unprepared to provide cross-cultural care [14]. The student perspective on the current state of training with regards to AsAm patients remains understudied. Students are important stakeholders and including their perspectives can inform and direct cultural humility training in medical education for the AsAm population. In this qualitative study, we aim to explore medical student perceptions on their motivation, preparation, and recommendations for improving cultural humility training related to caring for AsAm patients in undergraduate medical education in the US.

## Methods

An online survey, containing both open- and close-ended questions, was distributed to a convenience sample of nine medical schools across the US to assess medical students’ knowledge and attitudes regarding the care of AsAm patients [13]. Medical students who completed at least one clinical rotation were eligible to participate in the survey. Respondents provided written informed consent by completing the survey and received a $5 Amazon gift card for participating. Further details regarding the survey methods have been reported previously [13]. This study received approval by the Johns Hopkins School of Medicine Institutional Review Board (IRB00201533).

The present study focuses on the qualitative analysis examining medical student responses to optional free text questions presented at the conclusion of the survey: “How much more do you want to learn about Asian and AsAm health disparities and topics specific to these patients?” and “Any other thoughts about your preparation to care for these patients?” All participants who provided a response to the optional questions were included in the analysis [15]. All participants who provided a response to the optional questions were included in the analysis. Thematic analysis was conducted in an iterative, multi-step process [16, 17]. First, the responses were divided in half and three coders (HV, SML, TKL) independently created codebooks documenting emergent themes. The codebooks were compared to develop one preliminary codebook, with the majority voting on split decisions. A sample of twenty new un-coded responses was then trialed with this codebook among three coders (HV, SML, TKL), and inter-rater reliability was measured using the iota coefficient (iota = 0.889, indicating good fit) [18]. The same three coders then used this finalized codebook to code all the responses, and these codes were reviewed by another party (SP). The use of multiple coders, the establishment of intercoder agreement, and external peer auditing are established methods to confirm reliability of the qualitative analysis process [19].

## Results

**Fig.1:**
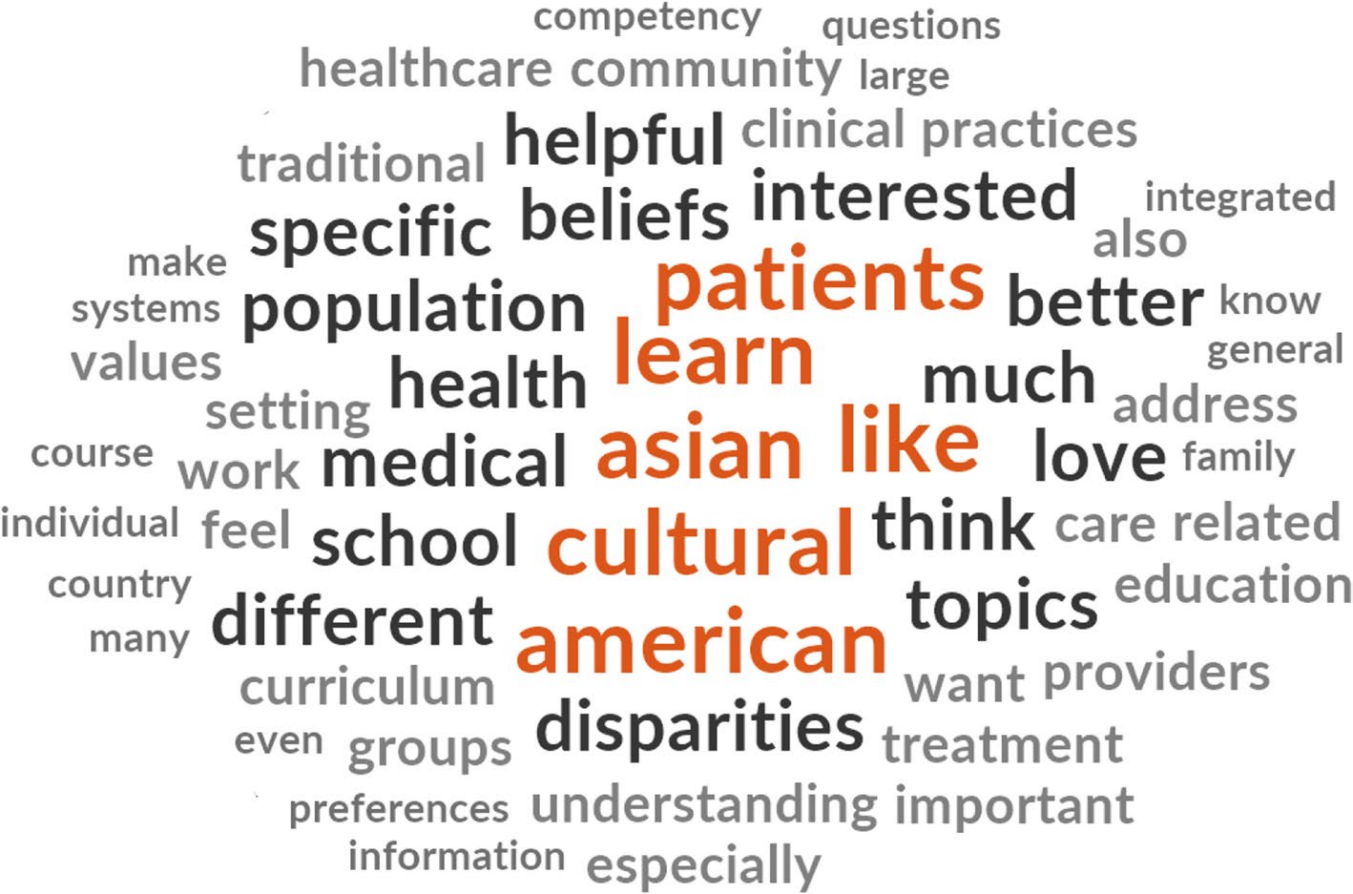
Word cloud with relative frequency of words represented by word size in open-ended responses

There was a total of 195 free-text responses from 688 participants (28%, Table 1). The median age of respondents was 25 (IQR: 25–27), with 105 (54%) females.The most reported race/ethnicity of participants was White (*n* = 99, 51%), followed by Asian (*n* = 84, 43%). In the surrounding county for each medical school, the AsAm population ranged from 4.8 to 39.7% [20]. There were 7 thematic categories identified: motivation, topics of interest, sources of knowledge, challenges, suggestions/strategies, disaggregation, and interest in learning more about diverse groups (Fig. 1). Representative quotes for each category were selected and are presented in Table 2.

**Table 1:** General characteristics of respondents included in qualitative analysis

Characteristic	Overall (*N* = 195)
Age (median, IQR)	25 (25, 27)
Gender	
Female	105 (54%)
Male	89 (46%)
Prefer not to answer	1 (0.5%)
Medical School^a^	
Baylor College of Medicine	24 (12%)
David Geffen School of Medicine at UCLA	12 (6.2%)
Harvard Medical School	33 (17%)
Johns Hopkins University School of Medicine	31 (16%)
Medical College of Wisconsin	20 (10%)
Northwestern University Feinberg School of Medicine	20 (10%)
Stanford University School of Medicine	12 (6.2%)
The Dell Medical School	11 (5.6%)
Washington University School of Medicine	32 (16%)
Reported race	
Asian	84 (43%)
White	99 (51%)
Black or African American	10 (5.1%)
Hispanic, Latino, or Spanish	14 (7.2%)
Middle Eastern or North African	7 (3.6%)
Native Hawaiian or Pacific Islander	2 (1.0%)
American Indian or Alaskan Native	3 (1.5%)
Not sure or don't know	3 (1.5%)

^a^Percent Asian population surrounding each medical school include Harris County (Baylor): 7.8%, Los Angeles County (UCLA): 16.3%, Suffolk County (Harvard): 9.8%, Baltimore City and Baltimore County (Johns Hopkins): 5.4%, Milwaukee County (Medical College of Wisconsin): 5.0%, Cook County (Northwestern): 8.3%, Santa Clara County (Stanford): 39.7%, Travis County (Dell): 8.1%, St. Louis City and St. Louis County (Washington University): 4.8%. Estimates were obtained from the American Community Survey 2019 5-year Estimates Table DP05, Asians alone or in combination with one or more races [20]. The estimates for St. Louis County and Baltimore County also include St. Louis City and Baltimore City respectively as the corresponding cities have their own separate jurisdiction and tabulate their demographic population separately from the county

**Table 2:** Themes and subthemes with representative quotes

**Theme**	Explanation	Exemplary Quotes
Motivation	Reasons that explain why students are driven to learn more about topics related to AsAm culture	
•Identity	Students share personal characteristics (e.g., race/ethnicity, culture, language, nation of origin) with AsAm patients	“I have had zero lecture time dedicated to discussing the culture and approach to taking care of Asian/Asian American patients. Being Asian American myself, this is very disappointing..” "I wish when we talk about cultural discrepancies in med school it's not just Black and Hispanic patients but also Asian patients. Somehow we are always left out of the conversation.”
•Service	Students hope to provide care for Asian/AsAm patients in their local community that meets their healthcare needs. They also feel that increased knowledge will have a positive impact on health outcomes	“There is a strong Hmong population where I attend medical school and we have not received adequate education as to their culture and general practices/preferences. This education would have been beneficial in many of my clinical rotations.” “Would like to learn about religious and traditional beliefs that may compliment or interfere with medical treatment.”
•Personal enrichment	Students want to learn more about AsAm culture because they want to increase their skills and knowledge in this area	“I think that in order to be a competent doctor, it is super important to understand my patients' culture-specific and individual-specific belief systems, cultural understanding, etc. Learning about this for any culture (including for Asian patients), could only help me to better serve the needs of my patients.” “I would love to learn to better communicate beyond my level of conversational Chinese.”
•Dissenting	Students disagree that increased knowledge or training related to AsAm culture would be beneficial to them	“In cities like my own, in which there is not a large Asian community, [training on these health disparities] probably would not make as much sense as it would for areas with a different population makeup.” “Have not had patient experience where their culture beliefs (apparent to me) impacted care, so a brief session would suffice.”
Topics of interest	Topics related to AsAm culture and healthcare disparities that students would be interested in learning more about	
•Cultural preferences regarding health and healthcare	Topics within AsAm culture that would allow students to better understand AsAm patients’ preferences or perspectives on Western medicine and healthcare (e.g. traditional medicine, ideas surrounding death and reincarnation)	“Stronger knowledge base of medical things that conflict with traditional beliefs would be helpful…” “Would be helpful to learn East and Southeast Asian thoughts on illness and death.”
•Healthcare delivery	How to best deliver culturally sensitive healthcare to this group (e.g. translation services, interviewing strategies)	“I would be interested to learn more about evaluating language proficiency in order to better utilize translation services.”
•Health outcomes/issues	How culturally sensitive care and other social factors affect clinical outcomes	“I would like to know about disparities fared by Asian Americans when socioeconomic status is taken into account.”
•Interest in learning more about diverse groups	Students reported interest in increasing knowledge about culture and preferences of minority patients in general	“I see patients from all different cultural backgrounds and I'd be interested in gaining more cultural competence across the board so I can be a better provider for all of my patients.”
Reported sources of knowledge	Experiences and/or events by which students have or want to learn more about AsAm culture	
•Personal	Personal experiences, background/identity, social/family network, and/or previous undergraduate classes or activities	“I’m only prepared so far as that I am drawing from my own individual Asian American identity and experience.” “The most valuable interactions were with the [local] Hmong population and also talking with my own family. I think having a Global Health background has certainly helped.” “I have learned a great deal more about Asian culture from making friends with classmates from China and India than I have in 3 years of medical school.”
•Pre-clinical exposures	Medical school classes and/or extracurricular activities	“We took an online course on health disparities in different cultures, including among different Asian cultures.” “Our education about AsAm health issues was limited to talks from APAMSA (a student organization).” “…assigned reading of ‘A Spirit Catches You and You Fall Down’ before the first year of medical school.”
•Clinical exposures	Patient and/or clinical staff interactions during clinical rotations	“Attendings that are able to divulge that information to students are some of the best.” “I study in an area with a much higher proportion of Asians than most areas of the country.”
Challenges	Challenges to learning about AsAm health topics and patient populations in medical school	
•Lack of exposure	Lack of exposure in both medical school curriculum and clinical experiences	“Asian/Asian Americans make up such a tiny proportion of the population we serve which I feel, unfortunately, contributes to my lack of knowledge or specific training regarding Asian and Asian American health.” “I feel that traditional medical school curricula, textbooks, and resources in general rarely address the Asian/Asian American population.”
•Other challenges	Other limiting factors	“I think it can be hard to become well-versed in various cultural differences, especially given different patient populations in different areas of the country.” “I also find myself tending to avoid picking up [AsAm] patients that require the use of an interpreter mainly because I do not know how to use the [interpreting] system”
Suggestions/strategies	Participant suggestions on how to incorporate AsAm-health related topics and cultural humility into medical education	
•Pre-clinical	Lectures, intersession courses, etc. during the pre-clinical curriculum	“Giving a crash course on some of these perspectives and then mainly teaching skills and strategies for making sure we appropriately address these concerns in our patients would be very helpful.” “Pre-clinical lectures that address this topic would be helpful before clinical years.”
•Clinical	Clinical sessions and educational resources	“I would like at least a couple of practice cases demonstrating cultural or religious issues that may arise while treating Asian patients.” “I would also like to have some hands-on practice with collaborating with a translator.” “I would love to see 'one page memos' with hyperlinks and short summaries that link to longer pieces of information.”
•Other suggestions for format	Longitudinal integration, mandatory, catering content to different institutions based on patient population	“At the very least, there should be seminars or similar sessions 1–2 times a year discussing the importance of cultural competency and specific health disparities for Asians and Asian Americans… The stress on cultural competency seems to have faded in third year and has not been a part of our non-clerkship didactics.” “Trainees should learn about the patients that they are likely to treat, so it may make more sense to have different curricula at different institutions.”
Disaggregation	AsAm are treated as a monolith in medical education, despite the heterogeneity that exists and should be addressed	“Asian American health disparities are highly variable and depend on which Asian country and how long they have been in the US. It is not a good idea to bin all Asians together.”

### Motivation

Participants cited a variety of motivating factors for learning more about AsAm patients and healthcare. Several students, who self-identified as Asian or AsAm, noted that having certain personal characteristics (e.g., race/ethnicity, language, culture, religion, or nation of origin) in common with AsAm patients inspired them to want to learn more about this group. Other participants who shared this identity also felt that the traditional medical curriculum often exclusively focuses on health care disparities affecting Black and Latino communities to the exclusion of AsAm communities. Some students wanted to learn more about AsAm culture and preferences because they wanted to be better equipped to meet their patients’ needs, particularly in local communities. In one example, a participant whose medical school has a large surrounding Hmong population, responded that they wanted to learn more about Hmong traditions and religious beliefs that may impact patients’ choices regarding medical treatment.

Another motivating factor was the desire for personal enrichment. One respondent felt that an increased understanding of patients’ belief systems and culture would make them a more competent physician. Conversely, not all participants felt that increased knowledge about Asian culture would be beneficial. Two students remarked that they did not see the value of additional training in this area because they did not attend school or plan to practice in a city with a large AsAm population. One student remarked that they had not had an experience where a patient’s cultural beliefs impacted the patient’s care.

### Sources of knowledge

There were varied responses from participants about the sources from which they drew their knowledge about AsAm culture and healthcare preferences. One common source was personal experience, including an individual’s background or upbringing, pre-medical school training or experiences, and family or social networks. Some students who self-identified as AsAm acknowledged their reliance on and the limitations of their own experiences to inform them of what may apply to their patients. Multiple participants looked to their AsAm classmates to learn more about their cultures.

Another source of knowledge was pre-clinical experiences during medical school. Students mainly reported that learning around this topic was largely self-initiated, including participating in cultural or affinity student organizations, attending faculty talks and panels, or searching for related research articles and conferences. There were few responses discussing specific medical school courses or assignments focused on AsAm culture and health disparities.

Clinical exposures were also cited as a source. Some participants recorded their interactions with AsAm patients in the hospital and clinic as possible sources of knowledge, but there were a few students who mentioned that they had little to no exposure to AsAm patients while on their clinical rotations. Only one participant highlighted their attending physician as a source of knowledge (Table 2).

### Topics of interest

AsAm cultural preferences/perspectives and cultural humility training regarding this population appeared to be a significant topic of interest for respondents. A recurrent suggestion was to provide more education about traditional Asian beliefs and practices surrounding medicine and healing.

Students acknowledged that a better understanding of specific cultural perspectives surrounding health and illness could help them better tailor treatment recommendations and related healthcare conversations with AsAm patients, and potentially improve clinical outcomes. Participants also noted that this understanding might bridge the barriers that prevent AsAm patients from seeking medical care or other resources, such as social work or physical therapy. While participants advocated for increased AsAm knowledge, participants also commented on the need for increased education and understanding of better cultural care for all minority patients. To better provide direct patient care, one student responded that they wanted to improve their Mandarin language skills.

### Challenges

Participants reported that there was a lack of, or limited coverage of AsAm health-related topics or cultural humility in medical school. Many participants noted that their medical schools’ surrounding patient population was generally lacking in AsAm representation or was predominantly made up of one subgroup, which limited their clinical and curricular experience with diverse AsAm cultures. On the other hand, a few participants mentioned that despite a large AsAm patient population near the school, curricular emphasis on AsAm was still limited. Without guidance or formal instruction, a few participants noted that they were unsure how to learn about AsAm health or had to find their own ways to supplement their learning. Some participants cited language barriers and not being certified interpreters as challenges they faced when caring for AsAm patients.

Other challenges that participants mentioned included the vast diversity within AsAm which made it difficult to adequately learn or teach about this population and limited time in the curriculum to be able to cover these topics. A few participants noted that stereotypes (e.g., model minority myth) were the cause of the lack of discussion of AsAm groups in healthcare. When AsAm groups were discussed in the curriculum, the model minority myth was perpetuated as AsAms were viewed as a relatively healthy minority group.

### Suggestions

Participants raised several suggestions for incorporating AsAm health-related and cultural humility topics into their medical education. In the pre-clinical curriculum, participants requested dedicated time for AsAm topics, whether this took form in formal didactics, integration into existing courses, intersession courses, or seminars, depending on a school’s curricular structure. Possible sessions include speaker panels, skills sessions, and patient cases.

During the clinical curriculum, students wanted more hands-on learning, such as practice cases, learning to work with interpreters, and opportunities for clinical interactions with AsAm communities. Participants also thought that a short summary document or pocket tool linking to additional resources that they could refer to and review on their own would be useful.

Notably, participants indicated that they wished for these topics to be integrated longitudinally throughout the pre-clinical and clinical curriculum. These topics should be taught in conjunction with discussion about other minority groups and should encompass all AsAm subgroups. Additional considerations that participants raised included making these topics mandatory in medical school and catering the content to different institutions based on the surrounding AsAm patient population. A few participants expressed that a brief session or 1–2 lectures would suffice, as there is limited space in the medical curriculum.

### Disaggregation

Disaggregation was a common thread throughout all the previous themes discussed. Participants noted the heterogeneity of the Asian population (e.g. different subgroups, underrepresented minorities, first- versus third-generation immigrants, etc.), and how this is both a priority and challenge in understanding and advocating for this population within medical education. When included in curricula, AsAms are treated as a monolith, not accounting for the diversity within the heterogeneous population. Participants further reported that this diversity makes it difficult to engage in a one-size-fits-all approach for AsAms and necessitates a thoughtful approach in how to respect the unique cultural values and features that may be present in different Asian ethnic subgroups.

## Discussion

In this study, US medical students expressed interest in learning more about AsAm culture and health disparities in their medical education, but highlighted the gaps in their current medical school curricula. To our knowledge, this is the first qualitative study on this topic in the medical education literature.

Students were motivated to learn about the AsAm population for many reasons. Some students felt connected to this population because of personal identity and background, and, importantly, others noted that improved training would better patient care. Despite student interest in this topic, participants reported a lack of curricular focus on AsAm health and health disparities in their medical education. This may reflect consequences of the “model minority myth” that emerged in the 1960s and the 1985 Heckler Report on Black and Minority Health that aggregated all Asian and Pacific Islanders and reconstructed public opinion that all AsAms are wealthy, hardworking, and, most importantly, healthy [21–23]. With the rise of anti-Asian hate during the COVID-19 pandemic, there is an increased need to understand the healthcare issues that affect the diverse groups within this population [24, 25]. The lack of this awareness, especially at the medical education level, limits change for the AsAm population.

### Recommendations & implications

We noted that that our participants had specific recommendations that targeted the differing Asian subgroups in the community around each medical school (for example, the Hmong population in Madison, Wisconsin). Participants in our study also recognized the vast diversity of AsAm subgroups and further acknowledged this diversity as a challenge for learning about and caring for AsAm populations. Indeed, patients identifying in different AsAm subgroups have different healthcare experiences [26, 27], aligning with our participants’ concerns that AsAm subgroups cannot be treated similarly. Additionally, in their responses, participants commonly requested education and training in cultural humility and health disparities for AsAms along with other minority groups. Despite the diversity that exists between minority groups, there are shared issues that minority patients face, such as language barriers, discrimination, and lower satisfaction with care [28].

Taken together, the heterogeneity within the AsAm population and shared healthcare challenges faced by various minority groups point to a general strategy to prepare medical students to treat AsAm patients. Rather than prescriptive, knowledge-based content of each AsAm subgroup or minority group, medical schools should identify the needs of the surrounding AsAm population and address them within their cultural humility curriculum. This may include educating students about how to utilize a certified medical interpreter, highlighting particular AsAm cultural practices in the surrounding community, or identifying surrounding community-based organizations that AsAm patients can be referred to. Including these practical recommendations into current medical school curricula will help students nurture active skills that can be widely used to better care for their AsAm patients.

In addition to this targeted approach, medical schools should also emphasize teaching cultural humility skills that can be applied broadly. This will allow students to best consider each patient’s individual background during their care, especially as participants noted that not all schools have exposure to the same AsAm or minority populations. Students are also likely to practice in a different geographical area than where they train for medical school, so equipping them with the skills to learn further on their own will be necessary. This curricular reform is already in development, as medical schools are moving towards a more skills-based cultural humility curriculum and focusing on broadly applicable skills, such as patient-centered care and communication [10]. Including AsAm representation in this curriculum, along with other minorities, is an important step in ensuring that students are prepared to apply these skills for patients of all backgrounds.

Students provided specific topics and suggestions on how to shape the curriculum to increase exposure to AsAm-related topics. Given the wide variation in cultural competency curricula [29], student input on topics and format of training can be helpful in moving towards a curriculum that is effective and specific to students’ circumstances and needs. In our study, participants requested opportunities to learn about AsAms in both preclinical and clinical training, including interactive activities like panels, simulation cases, and community experiences. These suggestions mirror the movement towards longitudinal and experiential integration of cultural humility training [29] and correlate with previous findings that fourth year students feel inadequately prepared in providing cross-cultural care [14]. One notable recurring topic was the lack of exposure regarding traditional medicine and non-Western perspectives on healing. In addition to helping medical students to understand certain AsAm cultural views, introducing these ideas may encourage medical students to expand their “worldview” that may hold implicit and explicit assumptions about health and the practice of medicine [30]. Medical schools should introduce curricula that help students develop more confidence in working with patients that seek out or use complementary and alternative therapies, such as acupuncture or herbal medicine.

### Limitations

There are some limitations to this study. Participants included in this qualitative analysis were volunteers who opted to respond to the optional survey question. Thus, these respondents may have greater interest or thoughts regarding AsAm topics in medical education compared to the general medical student population. While our respondents emphasized the necessity of recognizing the diversity of cultures and histories of the heterogeneous AsAm populations within medical education, our study asked respondents to consider AsAm in aggregate. Participants may have had differing comfort levels with and conceptions of different AsAm subgroups, for example Asian Indian versus Korean. Due to the relative lack of literature on this topic and the differing AsAm populations around medical schools throughout the US, we chose to utilize wording in this study that asked about AsAms in aggregate. Future qualitative work could focus on AsAm ethnic subgroups, particularly the unique communities that surround individual medical schools.

Furthermore, participants were selected from a convenience sample of nine medical schools, so our findings may not be representative of medical students nationally. However, the large number of responses and congruence of responses from students at different medical schools around the nation support the validity of our themes—our findings represent current student perspectives regarding AsAm cultural humility curriculum in medical education. Future work should include the perspectives of other important stakeholders, including patients, medical school administrators, and faculty at the local and national level, to understand barriers and facilitators to including cultural humility training regarding AsAm patients.

In conclusion, this study explored medical students’ attitudes about and recommendations for cultural humility training in medical school for AsAm populations. Medical students identified a need for greater inclusion and engagement of AsAm topics in medical education on cultural humility and minority health. We recommend that medical schools design longitudinal, experiential learning that considers the surrounding AsAm community around their respective medical school, as well as having AsAm representation in curricula on cultural humility. The result of our qualitative analysis contributes to ongoing work to improve cultural humility training in medical schools for patients of all backgrounds. Deliberate curricular reform with AsAm representation in mind will help future medical providers care for the diverse and growing AsAm population.

## Data Availability

The datasets used and/or analyzed during the current study are available from the corresponding author on reasonable request.

## Abbreviations

AsAm: Asian American
US: United states
